# Smartphone-based immersive virtual reality for pediatric preoperative preparation: development and initial feasibility report

**DOI:** 10.1016/j.bjane.2026.844825

**Published:** 2026-09-12

**Authors:** Andreia Cardoso, Durval Campos Kraychete

**Affiliations:** a Anesthesiologist Mater Dei Hospital, Salvador, BA, Brazil; b Universidade Federal da Bahia, Salvador, BA, Brazil; c Universidade Federal da Bahia (UFBA), Department of Anesthesiology and Surgery, Salvador, BA, Brazil

*Dear Editor*,

Preoperative anxiety affects 40% to 60% of children undergoing anesthesia,^1^ representing an important challenge, as it may affect anesthetic management and clinical outcomes.^2^ Unfamiliar surroundings, combined with perceived loss of control and uncertainty regarding medical procedures, may contribute to negative expectations and anticipatory distress.^2^ Preoperative preparation and other non-pharmacological approaches reduce anxiety and improve the experience of patients and caregivers.^1^ Among these, immersive Virtual Reality (VR) has emerged as a promising tool, with benefits attributed to two mechanisms: cognitive distraction and familiarization.^3^

In Brazil, accessible VR materials designed for pediatric preoperative preparation remain scarce. To bridge this gap, a multidisciplinary team developed a tailored immersive tool. This Letter reports the development and initial operational feasibility of the intervention within a prospective, single-arm exploratory pilot study conducted as part of the first author’s master’s research. The present report does not assess the effect of the intervention on preoperative anxiety, anesthetic outcomes, or postoperative recovery. The remaining outcomes of the pilot study will be reported in the master's dissertation and its derived publication, which will cite this Letter and will not duplicate the data presented here. During content planning, priority was given to determining the type and amount of information conveyed to the child, to promote comprehension without cognitive overload. The video reproduces the child's actual path through the facility, providing realistic and sequential exposure to surgical environments in age-appropriate, playful and reassuring tone. Safety was also considered throughout the design process. Although literature suggests limited evidence of harm from short, supervised exposure,^4^ potential discomfort and adverse effects remain relevant. Therefore, length and pacing were optimized to balance clarity and tolerability. Excessively long sequences could lead to boredom and disrupt clinical workflow, whereas an accelerated pace could limit immersion and predispose users to cybersickness symptoms, such as dizziness, nausea, visual disturbances, and headache. A structured description of the intervention following the TIDieR framework^5^ is provided as Supplementary Material 1.

Entitled *Viagem ao Centro Cirúrgico* [Journey to the Operating Room], the video combines real hospital footage with animated elements, structured as a spaceship voyage. A first-person corridor segment recorded with a drone simulates dynamic movement through the surgical center. Illustrations, such as fish swimming in scrub sinks and floating paper airplanes, were then incorporated to create a child-friendly scenario. A digital spaceship overlay was added, giving the illusion that the participant is a passenger traveling inside a cockpit, without compromising the realism of the original footage. The flight ends on the operating table, where audio narration invites passengers to disembark, and the perspective then shifts to a 360-degree live-action segment of the operating room. The final version is a passive, non-interactive video in Portuguese, 2 minutes and 51 seconds in duration, comprising a conventional first-person drone sequence followed by a monoscopic 360° equirectangular live-action segment, finalized in July 2024. It is permanently deposited in an open-access repository (doi: 10.5281/zenodo.21312318) and freely available on YouTube (www.youtube.com/watch?v=o3S77cTsIy4), playable on regular smartphones and simple VR viewers (Fig. 1).

**Figure 1:**
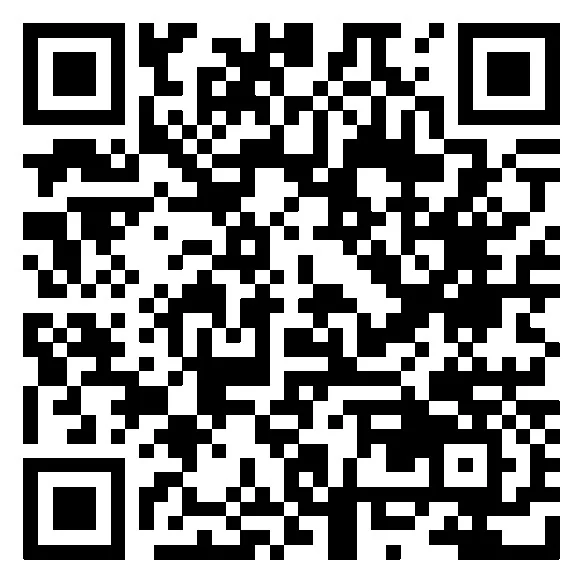
QR code providing access to *Viagem ao Centro Cirúrgico* [Journey to the Operating Room], the smartphone-based immersive virtual reality video developed for pediatric preoperative preparation. Scanning the code opens the video on YouTube. (https://www.youtube.com/watch?v=o3S77cTsIy4).

Between February and August 2025, the intervention was offered to children aged 5 to 12 years, ASA physical status I or II, in the admission area across two units of a private institution in Brazil. Exclusion criteria were neurodevelopmental disorders, history of seizures, visual or auditory impairment, regular use of psychoactive medication, and need for preoperative anxiolytics. Legal guardians provided written informed consent and children provided assent. The study was conducted in accordance with institutional ethical standards and approved by the local Research Ethics Committee (CAAE 59980922.9.0000.5128).

For each session, the child remained seated in an armchair while the video was displayed on a smartphone (Samsung Galaxy S21) inserted into a VR Shinecon headset and played through the YouTube application in VR mode. Audio was delivered through Bluetooth headphones integrated into the headset. All sessions occurred under direct anesthesiologist supervision. Adverse effects were monitored through continuous direct observation, with a prespecified criterion to interrupt playback upon any sign or spontaneous report of discomfort. No standardized symptom instrument was applied. Application time was measured from initial headset positioning to the end of the first complete viewing. The setup used an existing smartphone and a VR headset purchased for R$ 180 (approximately US$ 33). The intervention was integrated into the routine preanesthetic contact in the admission area, requiring no additional dedicated staff.

Of 23 children assessed for eligibility, three were not enrolled: one had received premedication, one was receiving regular psychoactive medication and one declined participation. Therefore, 20 were enrolled, corresponding to a recruitment rate of 87% (20/23). Mean age was 8.2 ± 2.0 years (range, 5.2–12.4), 12 (60%) were boys, and 15 (75%) were classified as ASAI. Scheduled procedures were urological (30%), general (30%), otorhinolaryngological (25%), and orthopedic (15%). All 20 children completed the full viewing (100% intervention completion). There were no technical failures, interruptions, requests to remove the headset, and no observed or spontaneously reported adverse effects. Mean application time was 4 minutes 16 seconds (SD = 13s; range, 4 min 5s to 4 min 51s). No acceptability claims are made in this Letter, as no validated standardized acceptability questionnaire was administered.

The main contribution of this initiative is the materialization of a functional, freely accessible VR tool specifically developed for pediatric preoperative preparation in the Brazilian context. In this small single-arm sample, all enrolled children completed the intervention, and the mean application time was 4 minutes 16 seconds. These descriptive observations should not be interpreted as evidence of safety, efficacy, or broad acceptability. Although the per-unit equipment cost was low and the video is freely available, a relevant feature for access in the Brazilian context, no formal cost-effectiveness or implementation analysis was conducted, and these questions, along with anxiety-related outcomes, require evaluation in further studies. While evidence supports VR-based preoperative preparation in general,^3^ the evidence generated by this specific intervention is currently limited to the development process and initial feasibility experience reported here.

## Data availability statement

No new data were generated or analyzed in this article. Data sharing is not applicable to this article.

## Ethics approval

The study was approved by the local Ethics Committee (CAAE: 59980922.9.0000.5128).

## Study registration

Not applicable.

## Permissions

Hospital footage was recorded with written institutional authorization covering academic use and publication. All professionals involved in the production (filming, illustration, editing, and narration) provided written assignment of use rights covering academic publication and open-access deposit. The soundtrack is royalty-free.

## Authors’ contributions

Andreia Cardoso contributed to study conception and design, data collection, interpretation of findings, and drafting of the manuscript.

Durval Campos Kraychete contributed to project supervision, intellectual development of the study, critical revision of the manuscript, and final approval of the submitted version.

## Conflicts of interest

The first author and the audiovisual director hold a pending trademark application for *Viagem ao Centro Cirúrgico* at the Brazilian National Institute of Industrial Property (INPI). The intervention is distributed free of charge, and no commercial exploitation exists or is planned. The authors declare no other conflicts of interest.

## Appendix Supplementary materials

Image, application 1

## Footnotes

Supplementary material associated with this article can be found in the online version at doi:10.1016/j.bjane.2026.844825.

## References

[1] Capurso M., Ragni B. (2016). Psycho-educational preparation of children for anaesthesia: A review of intervention methods. Patient Educ Couns.

[2] Adler A.C., Leung S., Lee B.H., Dubow S.R. (2018). Preparing Your Pediatric Patients and Their Families for the Operating Room: Reducing Fear of the Unknown. Pediatr Rev.

[3] Koo C.H., Park J.W., Ryu J.H., Han S.H. (2020). The Effect of Virtual Reality on Preoperative Anxiety: A Meta-Analysis of Randomized Controlled Trials. J Clin Med.

[4] Bexson C., Oldham G., Wray J. (2024). Safety of virtual reality use in children: a systematic review. Eur J Pediatr.

[5] Hoffmann T.C., Glasziou P.P., Boutron I. (2014). Better reporting of interventions: template for intervention description and replication (TIDieR) checklist and guide. BMJ.

