# Supplementary material for: Smartphone-based immersive virtual reality for pediatric preoperative preparation: development and initial feasibility report
Source: Braz J Anesthesiol. 2026 Sep 12;76(6):844825. doi: 10.1016/j.bjane.2026.844825 (PMC13636335; doi:10.1016/j.bjane.2026.844825)
Supplement: Supplementary file 1 [file mmc1.docx]

**BJAN-D-26-00471_Supplementary Material**

**Supplementary Material 1**

**TIDieR Checklist**

**Intervention:** *Viagem ao Centro Cirúrgico* [Journey to the Operating Room] — smartphone-based immersive virtual reality for pediatric preoperative preparation.

| **#** | **TIDieR item** | **Description** |
| --- | --- | --- |
| 1 | **Brief name** | *Viagem ao Centro Cirúrgico* [Journey to the Operating Room]: a smartphone-based immersive virtual reality video for pediatric preoperative preparation. |
| 2 | **Why** | Preoperative anxiety affects 40–60% of children undergoing anesthesia. Immersive VR may reduce anticipatory distress through cognitive distraction and familiarization with the surgical environment. Accessible VR materials designed for this purpose are scarce in Brazil. |
| 3 | **What — materials** | A passive, non-interactive video (2 min 51 s; Brazilian Portuguese narration and soundtrack; finalized July 2024) combining real hospital footage with animated elements, structured as a spaceship voyage. Initial first-person corridor segment recorded with an FPV drone (CineLog 25 with GoPro Hero 11 Black); frame-by-frame rotoscoped animations (e.g., fish in scrub sinks, paper airplanes); digital spaceship cockpit overlay created in After Effects; invisible-cut transition to a single 360-degree live-action take of the operating room (Insta360 X3) showing staff in surgical attire. Optimized for YouTube and standard VR player applications. Permanent open-access deposit: doi 10.5281/zenodo.21312318; also on YouTube ([www.youtube.com/watch?v=o3S77cTsIy4](http://www.youtube.com/watch?v=o3S77cTsIy4)). Display equipment: smartphone (Samsung Galaxy S21 in this study) inserted into a VR Shinecon headset; audio via headphones integrated into the headset (Bluetooth) or, for the headset without integrated headphones, the smartphone speaker. On iOS devices, playback requires a free VR player application; a Cardboard-type viewer is a possible alternative. |
| 4 | **What — procedures** | Delivered in the surgical admission area before anesthesia, integrated into the routine preanesthetic contact. The child was seated in an armchair and instructed to remain seated. Before starting: image focus, audio volume, and headset fit were checked. The video was shown in full; the child could watch it again on request. The headset was disinfected after each use with a disposable wipe soaked in 70% alcohol. |
| 5 | **Who provided** | An anesthesiologist (the researcher or a staff anesthesiologist previously trained through standardized practical sessions and written materials). |
| 6 | **How** | Individually, face to face, one child per session, under continuous direct supervision. |
| 7 | **Where** | Admission areas of two units of a private hospital network in Brazil (Belo Horizonte and Salvador), with comparable physical environment and care workflow. |
| 8 | **When and how much** | Once, on the day of surgery, before anesthesia and before any premedication. Mean application time 4 min 16 s (SD 13 s; range 4 min 5 s–4 min 51 s), measured from initial headset positioning to the end of the first complete viewing; spontaneous repeat viewings not included. |
| 9 | **Tailoring** | None. The intervention was standardized; the only allowed variation was optional repeat viewing at the child's request. |
| 10 | **Modifications** | None during the study period. |
| 11 | **How well — planned** | Fidelity supported by: standardized delivery procedure and pre-viewing checks; continuous direct observation by the supervising anesthesiologist with a prespecified criterion to interrupt playback upon any sign or spontaneous report of discomfort; structured recording of application time, completion, interruptions, technical failures, and adverse effects for every session. |
| 12 | **How well — actual** | All 20 enrolled children received the intervention as planned: 100% completed the full first viewing, with no interruptions, technical failures, or observed/spontaneously reported adverse effects. |

TIDieR, Template for Intervention Description and Replication (Hoffmann TC, et al. BMJ. 2014;348:g1687).

**Data Dictionary — Feasibility Dataset (de-identified)**

**Dataset:** DS_v1 (n = 20)

**Study:** *Viagem ao Centro Cirúrgico* — single-arm exploratory pilot study of smartphone-based immersive virtual reality for pediatric preoperative preparation. Rede Mater Dei de Saúde (two units, Brazil), February–August 2025. CAAE 59980922.9.0000.5128.

**De-identification:** direct identifiers (name, hospital record number, date of birth, date of data collection) and free-text procedure names were removed.

Age is provided in decimal years. Procedures are grouped into broad surgical categories.

Access is controlled owing to the small pediatric sample and re-identification risk.

| **Variable** | **Type** | **Values / units** | **Definition** |
| --- | --- | --- | --- |
| Participant Id | integer | 1–20 | Arbitrary sequential study identifier assigned for data management. Not linkable to hospital records or chronological order of enrollment. |
| Age (years) | numeric | decimal years | Age at intervention, computed from completed years and months (e.g., 5 y 2 m = 5.17). |
| Sex | categorical | female, male | Biological sex as recorded in the clinical chart. |
| ASA status | categorical | I, II | American Society of Anesthesiologists physical status classification. |
| Surgical Category | categorical | General, Urological, Otorhinolaryngological, Orthopedic | Broad category of the scheduled elective procedure, per surgical team. |
| Application Time (mm:ss) | text | m:ss | Total application time: from initial VR headset positioning and child instructions to the end of the first complete viewing of the 2 min 51 s video. Spontaneous repeat viewings not included. |
| Completed Full Viewing | categorical | yes, no | Whether the child watched the video in its entirety (first viewing). |
| Interruption | categorical | yes, no | Any interruption of playback or request to remove the headset. |
| Technical Failure | categorical | yes, no | Any playback, audio, image, connection, or device-adjustment failure, or operational interruption. |
| Adverse Event | categorical | yes, no | Any observed or spontaneously reported VR-related symptom or discomfort during the intervention, including nausea, dizziness, headache, visual disturbance, or headset-related discomfort. |

**Supplementary Material**

| **Participant Id** | **Age (years)** | **Surgical Category** | **Sex** | **ASA Status** | **Application Time** | **Completed full viewing** | **Interruption** | **Technical Failure** | **Adverse Event** |
| --- | --- | --- | --- | --- | --- | --- | --- | --- | --- |
|  |  |  |  |  |  |  |  |  |  |
| 1 | 5.17 | General | Female | I | 4 min 16s | Yes | No | No | No |
| 2 | 7.0 | General | Female | I | 4 min 17s | Yes | No | No | No |
| 3 | 9.33 | Urological | Male | I | 4 min 12s | Yes | No | No | No |
| 4 | 6.58 | Otorhinolaryngological | Male | I | 4 min 05s | Yes | No | No | No |
| 5 | 6.42 | Orthopedic | Male | II | 4 min 16s | Yes | No | No | No |
| 6 | 10.0 | Urological | Male | II | 4 min 05s | Yes | No | No | No |
| 7 | 5.67 | Otorhinolaryngological | Female | I | 4 min 08s | Yes | No | No | No |
| 8 | 7.17 | Urological | Male | I | 4 min 26s | Yes | No | No | No |
| 9 | 7.75 | General | Female | II | 4 min 09s | Yes | No | No | No |
| 10 | 7.42 | Otorhinolaryngological | Male | II | 4 min 16s | Yes | No | No | No |
| 11 | 11.67 | General | Female | I | 4 min 22s | Yes | No | No | No |
| 12 | 8.92 | Otorhinolaryngological | Male | I | 4 min 11s | Yes | No | No | No |
| 13 | 9,50 | Urological | Male | I | 4 min 51s | Yes | No | No | No |
| 14 | 12.42 | Urological | Male | I | 4 min 17s | Yes | No | No | No |
| 15 | 9.17 | Urological | Male | I | 4 min 12s | Yes | No | No | No |
| 16 | 9.33 | Orthopedic | Male | I | 4 min 5s | Yes | No | No | No |
| 17 | 5.67 | General | Female | I | 4 min 05s | Yes | No | No | No |
| 18 | 9.33 | General | Male | II | 4 min 06s | Yes | No | No | No |
| 19 | 8,08 | Orthopedic | Female | I | 4 min 07s | Yes | No | No | No |
| 20 | 6,42 | Otorhinolaryngological | Female | I | 4 min 08s | Yes | No | No | No |
